# Human brucellosis among pyrexia of unknown origin cases and occupationally exposed individuals in Goa Region, India

**DOI:** 10.3402/ehtj.v7.23846

**Published:** 2014-04-22

**Authors:** Ajay D. Pathak, Zunjar B. Dubal, Swapnil Doijad, Abhay Raorane, Savio Rodrigues, Rajeshwar Naik, Shraddha Naik-Gaonkar, Dewanand R. Kalorey, Nitin V. Kurkure, Rajesh Naik, Sukhadeo B. Barbuddhe

**Affiliations:** 1 Department of Veterinary Public Health, ICAR Research Complex for Goa, Old Goa, India; 2 Department of Microbiology, Goa Medical College, Bambolim, India; 3 Royal Hospital, Madgaon, India; 4 Department of Microbiology and Animal Biotechnology, Nagpur Veterinary College, Maharashtra Animal and Fishery Sciences University, Nagpur, India

**Keywords:** brucellosis, human, India, serology, ELISA, culture, PCR

## Abstract

**Background:**

Brucellosis is a widespread zoonotic infection. This disease is endemic in many parts of Asia, including India. Brucellosis is a major cause of pyrexia of unknown origin (PUO). Persons exposed to infected animals or contaminated animal products are at high risk. Seropositivity among animal handlers, veterinarians and dairy workers has been documented in India. Thus, the present study was aimed to determine prevalence of brucellosis among PUO cases and occupationally exposed individuals.

**Methods:**

In this study, serum samples (*n*=282) from cases of pyrexia of unknown origin (PUO) (*n*=243), and occupationally exposed individuals (*n*=39) were collected and tested for brucellosis by Rose Bengal plate test (RBPT), serum agglutination test (SAT), indirect ELISA, IgG and IgM ELISA. Blood culture for isolation of *Brucella* was performed for 10 serologically positive patients using BACTEC 9050 automated blood culture system. Biochemical tests and PCR techniques were used for confirmation of the isolates.

**Results:**

Of the samples tested, 4.25%, 3.54%, 6.02% and 4.96% samples were positive by RBPT, SAT, indirect ELISA and IgG ELISA, respectively. None of the sample was positive for IgM ELISA. Of the 10 blood samples cultured bacteriologically, one *Brucella* isolate was recovered. The isolate was confirmed as *Brucella abortus*. Amplification of the *bcsp31* and *IS711* genes was also observed.

**Conclusion:**

Seropositivity for brucellosis was observed among PUO cases, animal handlers and dairy workers in Goa, India. The serological tests showed variable results. One *Brucella* isolate was obtained by performing blood culture. Confirmation of the case was done rapidly using molecular tools. General awareness about clinical symptoms should be increased which will improve proper diagnosis within short time frame.

Human brucellosis is a major bacterial zoonosis reported worldwide. It is mainly an occupational disease reported in farmers, veterinarians, slaughterhouse workers, animal handlers and meat inspectors. It is caused by bacteria belonging to genus *Brucella*, which are small, Gram-negative, non-spore forming, non-encapsulated coccobacilli. More than 500,000 new cases are reported globally every year, with the annual incidence rates varying widely from <2 to >500 per 1,000,000 population among different regions (1). The disease remains endemic in many regions of the world including Latin America, the Middle East, Africa, Asia and the Mediterranean basin (1). International travellers visiting *Brucella* endemic regions are at high risk of infection. Transmission of *Brucella* to humans results from direct contact with the infected animal, as well as from consumption of unpasteurized milk and milk products (2, 3).

Human brucellosis is often misdiagnosed or underdiagnosed due to overlapping clinical manifestations with many bacterial infections. Undulant fever, weight loss and night sweats are the major symptoms of brucellosis in humans. It is one of the causes of fever of prolonged duration in endemic areas and one of the important causes of pyrexia of unknown origin (PUO) (4, 5). The pathogen causes abortion, infertility, retention of placenta, birth of weak and dead calves, and reduced milk yield in animals (6). Brucellosis cases can be classified as either probable or confirmed cases. A clinically compatible case linked epidemiologically to a confirmed case or having *Brucella* agglutination titre of greater than or equal to 160 in one or more serum specimens obtained after the onset of symptoms can be considered as a probable case (7). Laboratory confirmation of a clinically compatible case is a confirmed case. However, in brucellosis endemic countries, clinical symptoms associated with positive serology without isolation of *Brucella* spp. have been considered human confirmed cases (7).

The laboratory confirmation of human brucellosis is based on microbiological, serological or molecular methods, each having its own advantages and disadvantages. Many serological tests such as Rose Bengal plate test (RBPT), complement fixation test (CFT), Coombs test, ELISA, and serum agglutination test (SAT) are used for the diagnosis of human brucellosis (8–10). The molecular diagnosis of human brucellosis can be performed using genus-specific polymerase chain reaction (PCR) assays (11). Molecular assays targeting the *IS711* insertion element and the *bcsp31* gene, coding for a 31-kDa immunogenic outer membrane protein conserved among all *Brucella* spp. are the most common molecular targets in clinical applications (12, 13). Human brucellosis is usually diagnosed by agglutination based serological tests and ELISA, but isolation of the pathogen from blood culture remains the gold standard. The objective of the present investigation was to study the prevalence of brucellosis among cases of PUO and occupationally exposed individuals.

## Methods

### Sample collection

A total of 282 human blood samples from PUO cases (*n*=243) and occupationally exposed individuals (*n*=39) were obtained from Goa Medical College and Hospital, and other private hospitals from Goa, India. Serum was separated by centrifugation at 3,000 g for 10 min. Sera were stored at −20°C. Venous blood (8 mL) samples, which were positive in initial screening by SAT and RBPT, were obtained from 10 individuals for blood culture.

### Serological tests

#### Rose Bengal plate test

All the human sera were screened for brucellosis by RBPT. RBPT antigen was procured from Indian Veterinary Research Institute (IVRI), Izatnagar, India. The test was carried out according to standard procedure (14). Formation of agglutinate within 4 min with antigen was recorded as a positive reaction. Positive serum was obtained from *Brucella* Laboratory, IVRI, Izatnagar. Serum sample from a healthy individual was used as negative control. The positive and negative sera were used to compare the agglutination.

#### Serum agglutination test

All samples were tested by SAT according to standard procedure (14). *Brucella abortus* plain antigen (Phenol killed *Brucella abortus* S99) was procured from IVRI, Izatnagar, India. The highest serum dilution at which 50% agglutination was observed was marked as the end point for titre. A titre of 1:160 and more was regarded as serologically positive.

#### Enzyme linked immune-sorbent assay

Indirect ELISA was performed as described by Agasthya et al. (15). The smooth lipopolysaccharide (S-LPS) antigen of *Brucella abortus* S99 was procured from IVRI, Izatnagar. The serum collected from confirmed cases of brucellosis was used as strong positive serum control. The strong positive serum was obtained from *Brucella* Laboratory, IVRI, Izatnagar, which showed higher absorbance in ELISA. The serum sample taken from healthy individuals without any symptoms or history of animal contacts was used as the negative control serum. The moderate positive control was prepared by diluting the strong positive serum in negative serum (1:20 diluted). The rabbit antihuman HRP conjugate (Sigma), *o*-phenylenediamine dihydrochloride (OPD), hydrogen peroxide (H_2_O_2_), bovine gelatin (Sigma) and 96-well ELISA plate (Nunc polysorp) were used. Working dilutions of S-LPS antigen, control sera and rabbit antihuman HRP conjugate were established by a checkerboard titration for use in indirect ELISA. Control and test sera were used at 1:100 dilutions. A positive result was considered when the ELISA positive-negative ratio was ≥3.

IgG and IgM ELISA kits manufactured by Nova Tec Immunodiagnostica GmbH were used for IgG and IgM ELISA. The tests were performed according to manufacturer's instructions. The results were expressed in Nova Tec Units (NTU). A titre of 11 NTU or more was considered positive.

### Isolation and identification

Venous blood (8 mL) samples withdrawn from 10 serologically positive patients were inoculated directly into BD BACTEC™ PLUS-Aerobic/F Medium (BD Biosciences, Product code 442192) vials (30 mL) and processed using BACTEC 9050 automated blood culture system (Becton Dickinson Microbiology Systems, Sparks, MD, USA). Bacterial growth in the culture bottle was streaked on 5% sheep blood agar and *Brucella* agar with selective supplements (HiMedia Laboratories, Mumbai, India) and incubated in 5% CO_2_ atmosphere at 37°C. The plates were observed daily for evidence of growth. The plates were discarded after a week if no growth was observed. Small, round, white, non-haemolytic colonies on blood agar were subjected to identification by biochemical and molecular methods.

The isolates obtained were subjected to Gram staining, modified Ziehl-Neelsen staining, methyl red, Voges Proskauer, catalase, oxidase, urease, H_2_S production, sensitivity to dyes basic fuchsin (1:50,000) and thionin (1:50,000) (14).

For PCR, genomic DNA from bacterial culture was extracted by Phenol: Chloroform method. The quantity and purity of extracted DNA was determined by using Nano-Drop 1000 spectrophotometer (Thermo Scientific, Wilmington, DE, USA). The DNA was then subjected to PCR detection by primers specific to the *bcsp31* (B4/B5) and *IS711* genes (12, 16). Genomic DNA from *Brucella abortus* S19 was used as positive control, and DNA from *E. coli* ATCC 8739 was used as negative control.

The primers and other reagents were procured from Sigma Aldrich, Co., (St. Louis, MO, USA). The reaction mixture (25 µl) consisted of 12.5 µl of Ready Mix Taq buffer with MgCl_2_ (Sigma Aldrich, Co., St. Louis, MO, USA), 0.5 µl forward primer (10 pmole/ µl), 0.5 µl reverse primer (10 pmole/ µl), 10 µl of nuclease free water. To this mixture was added 1.8 µl of template DNA. For detection of *bcsp31* gene initial denaturation was done at 95°C for 3 min followed by 35 cycles of denaturation at 95°C for 45 s, annealing at 60°C for 45 s and extension at 72°C for 2 min. Final extension was performed at 72°C for 10 min. For detection of *IS711* gene, the PCR conditions were same as *bcsp31* except the primer annealing was done at 55°C. The PCR was performed using thermal cycler (Eppendorf Master Cycler, Germany). The PCR products were analysed by 1.5% agarose gel electrophoresis and were visualized using ethidium bromide staining under UV illumination (AlphaImager, USA).

## Results

Out of the 282 serum samples tested, 12 (4.25%) were positive for brucellosis by RBPT. A diagnostic titre of 1:160 was observed in 10 (3.54%) samples by SAT. Indirect ELISA detected 17 (6.02%) samples as positive. IgG titres were detected in 14 (4.96%) sera, but none of the sample demonstrated IgM titres. Of the 10 blood samples subjected to bacterial culture, one isolate was recovered using BACTEC automated blood culture system. The isolate was identified as *Brucella abortus* by performing biochemical tests and PCR.

Of the total samples tested, 25(8.86%) showed positivity to at least one test employed. Of the 12 RBPT positive samples, 10 (25.64%) samples were from the occupationally exposed group, which included animal handlers, dairy workers and veterinary workers. One PUO sample was found to be positive for RBPT. Surprisingly, one serum sample of a patient with no history of animal contact but manifesting PUO was found positive by RBPT. The patient had a history of frequent international travel to locations where brucellosis is endemic. In case of indirect ELISA, 14 (35.89%) samples were from the occupationally exposed group and 3 (7.69%) were of PUO cases. IgG ELISA was found positive in 11 (28.20%) occupationally exposed individuals, while 3 PUO cases were also found positive for this test.

*Brucella* agglutinins were reported in 12 cases by RBPT. RBPT positive samples demonstrated variable SAT titres; diagnostic titres were detected in only 10 samples. Indirect ELISA is a sensitive method of diagnosis. It demonstrated positive titres in 17 cases. The details are included in Table 1.

**Table 1:** Comparative results of serological tests and cultural isolation

Case no.	Case type	RBPT	SAT	Indirect ELISA	IgG ELISA	Blood culture
1	OE	–	1:80	–	–	ND
2	PUO	–	1:80	++	–	ND
3	OE	++	1:160	++	–	–
4	OE	–	1:80	++	–	ND
5	OE	–	1:80	++	–	ND
6	OE	–	1:160	++	–	–
7	PUO	–	1:80	++	–	ND
8	PUO	–	1:80	++	++	ND
9	PUO	–	1:80	–	–	ND
10	OE	++	1:160	++	–	–
11	OE	++	1:160	++	++	–
12	OE	++	1:160	++	++	–
13	PUO	++	1:160	–	++	–
14	OE	++	1:160	++	++	–
15	OE	–	1:80	++	++	ND
16	PUO	–	1:80	–	++	ND
17	OE	++	1:160	++	++	–
18	OE	++	1:160	++	++	–
19	OE	–	1:80	++	++	ND
20	OE	++	1:80	–	++	ND
21	OE	–	1:80	++	++	ND
22	OE	++	1:160	++	++	ND
23	OE	–	1:80	–	++	ND
24*	OE	++	1:80	–	–	+
25	PUO	++	1:80	–	–	ND

Note: –: negative; ++: positive; OE: occupationally exposed; PUO: pyrexia of unknown origin; ND: not done.

* : Culture positive case.

**Fig. 1:**
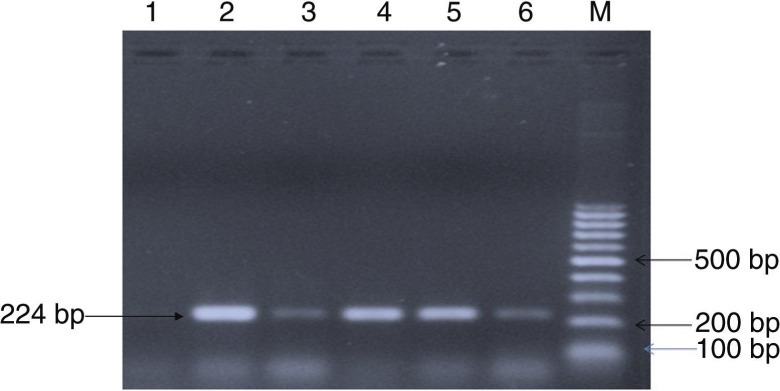
PCR amplification of the *bcsp31* gene. Lane 1: negative control; lane 2: positive control (*Brucella abortus* strain 19); lanes 3–6: *Brucella* isolate; M: 100 bp DNA ladder.

**Fig. 2:**
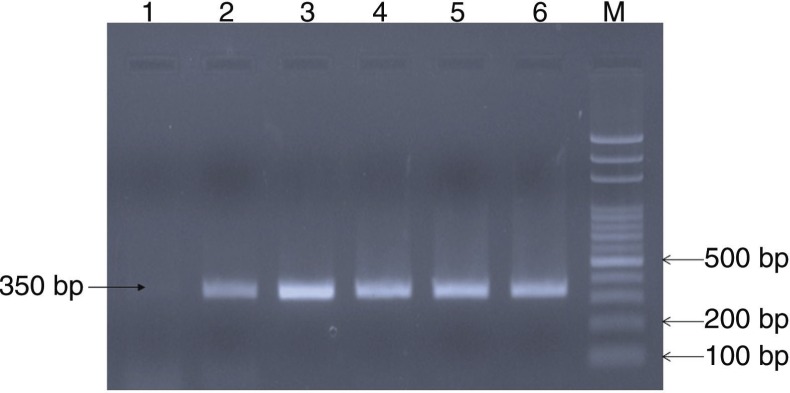
PCR amplification of the *IS711* gene. Lane 1: negative control; lane 2: positive control (*Brucella abortus* strain 19); lane 3–6: *Brucella* isolate; M: 1.5 Kb DNA ladder.

Isolation was attempted from 10 serologically positive cases (Table 1). Only one bacterial isolate was recovered after 72 hours of incubation at 37°C. The patient's serum sample was positive for RBPT and SAT but did not demonstrate significant titres in ELISA. The isolate showed tiny, white, non-haemolytic colonies on blood agar. The isolate was found to be positive for catalase, oxidase, urease, H_2_S production. Small Gram-negative coccobacilli in clumps were observed in Gram staining. Growth was observed on trypticase soy agar containing thionin (1:50,000) and basic fuchsin (1:50,000). Amplifications of the *bcsp31* (224 bp) and *IS711* (350 bp) genes were observed, which are universally accepted marker genes for detection of *Brucella* spp. (Figs. 1 and 2).

## Discussion

In the Indian subcontinent, brucellosis is prevalent among all domesticated animals and humans (17). Human brucellosis is acquired by contact with secretions of infected animals. Animal husbandry is the second largest occupation in rural India. Thus, the majority of the population has an increased risk of acquiring zoonotic infection. It is estimated that, due to underdiagnosis, the true incidence is 25 times higher than the reported cases (18).

In this study, seropositivity in 4.25%, 3.54%, 6.02% and 4.96%, samples was detected by RBPT, SAT, Indirect ELISA and IgG ELISA, respectively. In conjunction with a compatible clinical presentation, SAT titres above 1:160 are considered diagnostic (19); however, in endemic areas, a titre of 1:320 as cutoff may make the test more specific. In a similar study, Agasthya et al. (20) reported 2.26%, 2.26%, 19.69% samples positive by RBPT, SAT and Indirect ELISA, respectively among high-risk group individuals in Karnataka. Similarly, Mangalgi et al. (21) observed 4.79%, 4.41% and 4.41% seroprevalence among PUO cases by RBPT, SAT and 2ME, respectively in Bijapur, Karnataka. Also *Brucella* isolates were obtained from 26.4% cases. *Brucella* infection has been detected in 0.8 to 3.3% cases of PUO (22, 23).

Review of cases of brucellosis in Bangladesh revealed varied prevalence based on occupations of people (2.5–18.6%) (10). The prevalence of brucellosis was reported as 2.6–21.6% in livestock farmers, 18.6% in milkers, 2.5% in butchers and 5.3–11.1% in veterinarians who had direct contact with animals and their products or who consumed raw milk (10). The overall seroprevalence was found to be 6.9% among occupational groups in Pakistan (24).

The serological tests used in this study showed variable results. The most common clinical symptoms at presentation were fever, headache, low backache, arthralgia and myalgia. The disease was detected in an international traveller who was travelling between India and Mediterranean countries on a regular basis. The case was detected by RBPT and demonstrated titres in SAT and ELISA. Brucellosis has been reported as an important cause of travel-associated morbidity (25). Many travellers experience new foods, drinks and exotic food preparations and may get exposed themselves to many pathogens including *Brucella*, particularly when trying to get farm fresh ‘natural’ food.

None of the samples tested by IgM ELISA showed positivity indicating possible lack of active infection or chronicity of the disease. In an earlier report, the IgM ELISA did not show specific antibodies in 10 patients who were positive in conventional tests (26). Also, commercial IgM ELISA has a limited value in diagnosis of brucellosis due to low sensitivity (26). It has been opined that the variety of antigens used in the tests may lead to relevant differences among them. However, one *Brucella* isolate was obtained from a PUO patient without significant IgG and IgM titres. The isolate was obtained after 72 h processing in BACTEC 9050 blood culture system. Isolation of the pathogen was confirmed within 5 days by BACTEC blood culture method, which is significantly less time consuming than the classical biphasic culture technique.

It has been opined that genus-specific PCR can help to avoid false-negative results in patients infected with unusual species and biovars (22). In this study, we have used PCR targeting the *bcsp31* and *IS711* genes to detect the pathogen.

In conclusion, *Brucella* antibodies were detected in cases of PUO without a history of animal contacts and from occupationally exposed individuals. The serological tests showed variable results. The confirmation of cases of brucellosis indicated that awareness among clinicians can help to diagnose this disease within a short time frame. General awareness about the symptoms and prevention of brucellosis is warranted.
